# Where is the left ventricle during cardiopulmonary resuscitation based on chest computed tomography in the expiration with arms down position?

**DOI:** 10.1371/journal.pone.0193364

**Published:** 2018-02-23

**Authors:** Hyuksool Kwon, Yeokoon Kim, Kyuseok Kim, Jae Yun Jung, Joonghee Kim, Sang Il Choi, Eun Ju Chun, Woo Kyung Bae

**Affiliations:** 1 Department of Emergency Medicine, Seoul National University Bundang Hospital, Gumi-dong, Bundang-gu, Sungnam-si, Gyeonggi-do, Republic of Korea; 2 Department of Radiology, Seoul National University Bundang Hospital, Gumi-dong, Bundang-gu, Sungnam-si, Gyeonggi-do, Republic of Korea; 3 Health Promotion Center, Department of Family Medicine, Seoul National University Bundang Hospital, Gumi-dong, Bundang-gu, Sungnam-si, Gyeonggi-do, Republic of Korea; Public Library of Science, UNITED KINGDOM

## Abstract

**Objective:**

Patients usually receive cardiopulmonary resuscitation during ventilatory expiration and with their arms down, which does not reflect the normal imaging position. This study used scout images from low-dose chest computed tomography to compare the locations of the left ventricle (LV) in the expiration with arms down position (EAD) and in the full inspirational with arms raised position (IAR).

**Methods:**

This cross-sectional study used a convenience sample and evaluated scout images that were obtained during screening with the participants in the EAD and IAR positions. The effective compression point was defined as being on the sternum above the longest anteroposterior diameter (APD) of the LV (using axial computed tomography images). The sternum was divided into three parts and the heart’s position was evaluated on the EAD and the IAR images, and the distance from the xiphoid process to the LV’s sternum landmark (XLVD) was measured. We also examined the compressible organs during CPR based on the EAD and IAR images.

**Results:**

We enrolled 127 participants. The LVs were located in the middle of the sternum at EAD for 117 participants (92%) and in the lower half of the sternum at IAR for 107 participants (84%). The mean XLVD was significantly different between the EAD and IAR positions (mean: 85 ± 21 mm vs. 33 ± 17 mm, respectively). The liver’s left lobe was located in the lower half of the sternum at EAD for 118 participants (93%).

**Conclusions:**

These findings indicate that the location of the LV during cardiopulmonary resuscitation might be in the middle of the sternum if the patient is treated in the EAD position.

## Introduction

Chest compressions are essential for supplying blood to the vital organs during cardiopulmonary resuscitation (CPR). Hand position, compression depth, and compression rate are key determinants of high-quality CPR. However, the current CPR guidelines regarding the effective compression location for targeting the left ventricle (LV) have been derived from relatively few robust human data and a small number of studies, rather than assessing the compression rate and depth, to increase the quality of chest compressions during CPR [1–4].

Most evidence regarding optimal hand positioning for targeting the LV during compressions has been based on chest computed tomography (CT) or radiography [5–8]. However, this relationship is limited by the fact that patient positioning during chest CT or radiography can differ from patient positioning during real-life CPR. For example, to obtain CT images with fewer artifacts, patients are usually asked to perform deep inspiration and raise both arms during the imaging. However, full inspiration with the arms raised (IAR) does not accurately reflect real-life CPR, and expiration with the arms down (EAD) may more accurately reflect real-life CPR, because most patients are in the supine position with their arms down and have minimal muscular tone in their diaphragm and intercostal muscles. Furthermore, the heart and sternum move upward during expiration and downward during inspiration, along with diaphragm and chest wall movements, and the sternum and thoracic cage move upward when the arms are raised. Therefore, during conventional chest CT in the IAR position, the heart, diaphragm, and liver are moved downward and the sternum is moved upward [9]. These facts suggest that the location of LV might be more cephalad, rather than toward the lower half of the sternum, as CPR is typically performed with the patient in the EAD position.

The present study aimed to compare of the LV’s location in the EAD and IAR positions using low-dose chest CT (LDCT) findings in healthy volunteers underwent health screenings, not in patients undergoing CPR. We also evaluated the compressible organs during CPR based on the EAD and IAR images.

## Methods

### Study design

This cross-sectional study examined a convenience sample and was designed to evaluate the LV’s location during CPR based on LDCT images from health screenings. All participants provided informed consent for the data collection and the publication of their images, and the study’s protocol was approved by our institutional review board (B-1507/306-308).

Because the end of the xiphoid process was not identifiable in the scout images, a radio-opaque marker was attached to the skin over the xiphoid process to monitor changes in sternal location according to the positional changes. The xiphoid process marker was attached by a trained nurse, who performed palpation of the sternum’s distal end. Two scout images of the chest were obtained in the different positions before the LDCT for all participants. The first scout image was obtained in the EAD position to simulate real CPR positioning, and the second scout image was obtained in the IAR position, which is the standard position for LDCT. All LDCT procedures were performed in the IAR position only using a 128-slice multi-detector row CT unit (Ingenuity CT; Philips Medical Systems, Best, The Netherlands). The institutional picture archiving and communication system (G3 PACS; Infinite Inc., Seoul, Korea) was used to analyse the images for this study. All images were evaluated and interpreted by two board-certified chest radiologists.

### Inclusion and exclusion criteria

Consecutive participants who underwent LDCT during health screening between August 1 and December 31, 2015, were considered eligible. The inclusion criteria were an age of >18 years and no history of chest or abdominal surgery. The patients who had abnormality in chest that might have influenced respiration and who did not consent to participate were not enrolled. The patients whose radio-opaque marker was unidentifiable because of disturbing opacity of bones were excluded from analysis.

### Sample size

In a previous study of hand positioning for effective chest compressions during adult CPR [10], the proportion of distance from the xiphoid process to the sternal point at the longest outer anteroposterior diameter (APD) of LV compared to the length of the sternum was 13.6±7.3%. The required sample size of 110 was calculated based on the assumption that the distance from the xiphoid process APD of LV was 17±5% in the EAD position (25% more cephalad than the IAR position, a power of 80%, α of 0.05). The target sample size was increased by 15% to 127 to overcome a possibly higher standard deviation.

### Determining the point of the LV’s maximal APD using the IAR axial images and applying this point to the EAD scout images

**Fig 1:**
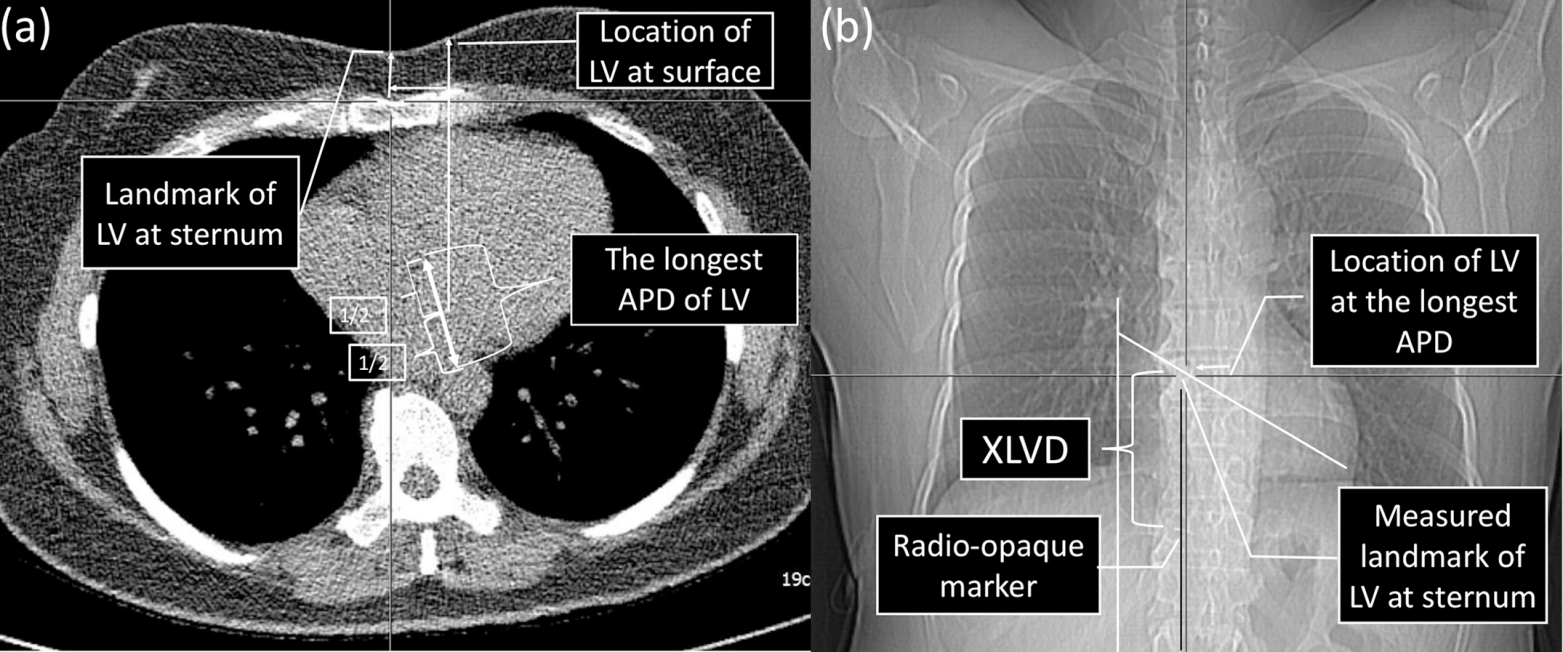
Determining maximal anteroposterior diameter (APD) of the left ventricle (LV) using axial and scout images. The skin above the maximal APD was searched to identify the sternum landmark representing the LV.
The maximal APD of the LV was measured and aligned in the axial images from the full inspiration with the arms raised position. The centre of the line was defined as the location of the LV (a).The location of the LV was marked at the skin and a perpendicular line was drawn to the sternum for the sternum landmark of the LV.A perpendicular line was drawn at the margin of the right atrial contour, and the intersection between the perpendicular line and the right atrial contour was marked.A straight line was drawn from the heart’s apex to the intersection point at the right atrial contour.Another perpendicular line was drawn from the radio-opaque marker at the lowest palpable xiphoid process to the line that connected the apex and intersection point. This intersection was defined as the LV sternum landmark for the expiration with the arms down position, and the distance from the sternum landmark to the xiphoid process marker was defined as the XLVD. This method was also applied to the expiration with the arms down position scout images.

We developed a method to identify a sternal location that reflected the LV’s maximal APD in the EAD position using the LV’s maximal APD from the axial CT images of the IAR position (Fig 1a). The LV’s maximal APD was measured on the axial CT images from the IAR position, and the LV’s sternal landmark was defined for the LV’s maximal APD on the EAD images (Fig 1b):

1. The LV’s maximal APD was drawn and measured on the axial CT images from the IAR position. The centre of the line was defined as the location of the LV.
2. The LV’s location was marked at the surface and a perpendicular line was drawn to the sternum for representing the LV’s sternal landmark.
3. A perpendicular line was drawn at the margin of the right atrial contour, and the intersection between the perpendicular line and the right atrial contour was marked.
4. A straight line was drawn from the heart’s apex to the intersection point at the right atrial contour.
5. Another perpendicular line was drawn from the radio-opaque marker at the lowest palpable xiphoid process to the line that connected the apex and intersection point. This intersection was defined as the LV’s sternal landmark for the EAD position, and we calculated the distance from the xiphoid process marker to the LV’s sternal landmark (XLVD).

**Fig 2:**
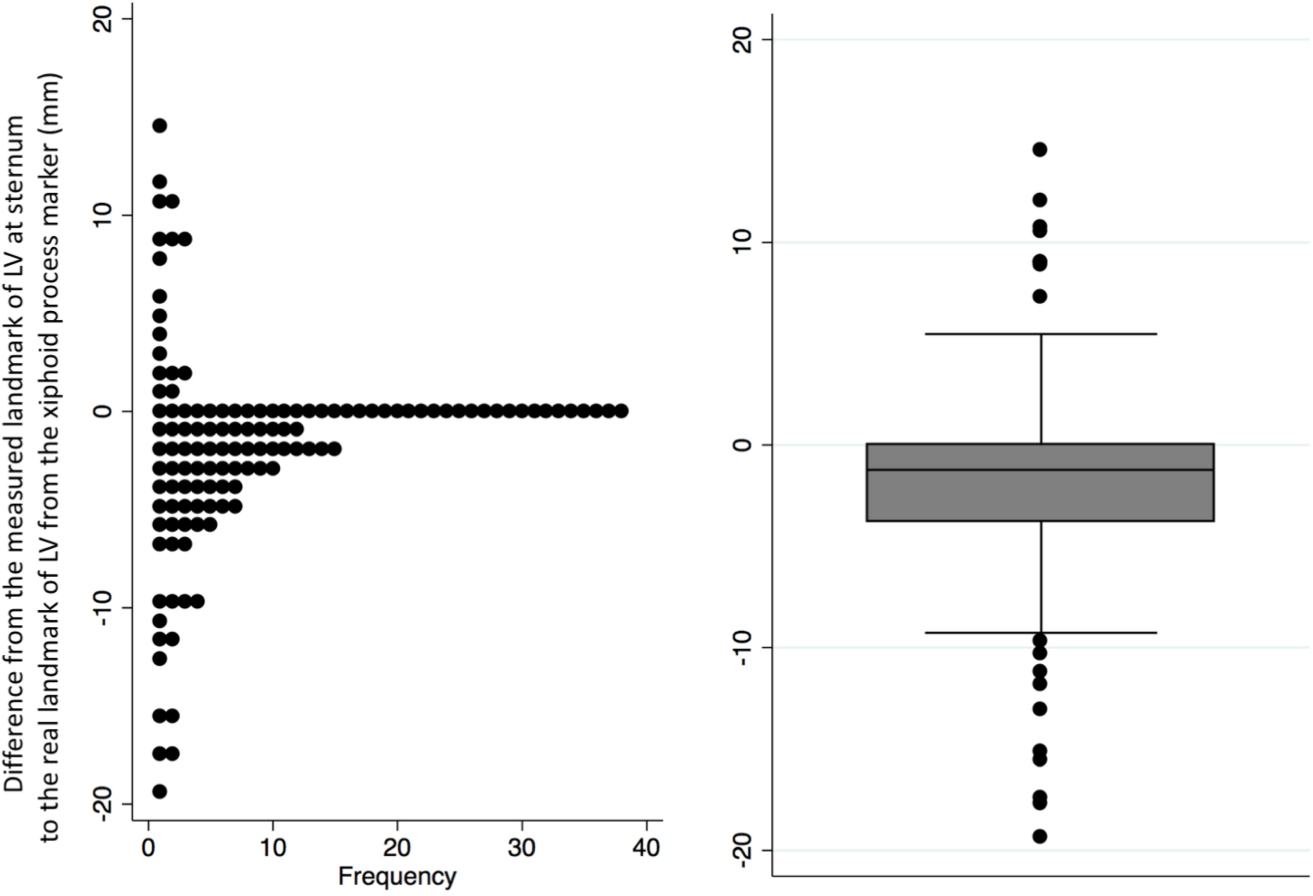
Distances from the arbitrary sternum left ventricle (LV) landmark to the actual landmark using axial images from full inspiration. The measured LV landmark was approximately 1.23 mm lower on the sternum, compared to the actual landmark (median: –1.23 mm, interquartile range: –15.58 mm, 10.56 mm, range: –19.35 mm, 14.52 mm).

The measured LV landmark from the IAR position was slightly lower than the actual LV landmark on the axial CT images (median: –1.23 mm, interquartile range: –15.58 mm, 10.56 mm, range: –19.35 mm to 14.52 mm) (Fig 2). Based on the relatively small difference, this method was applied to the EAD scout images.

**Fig 3:**
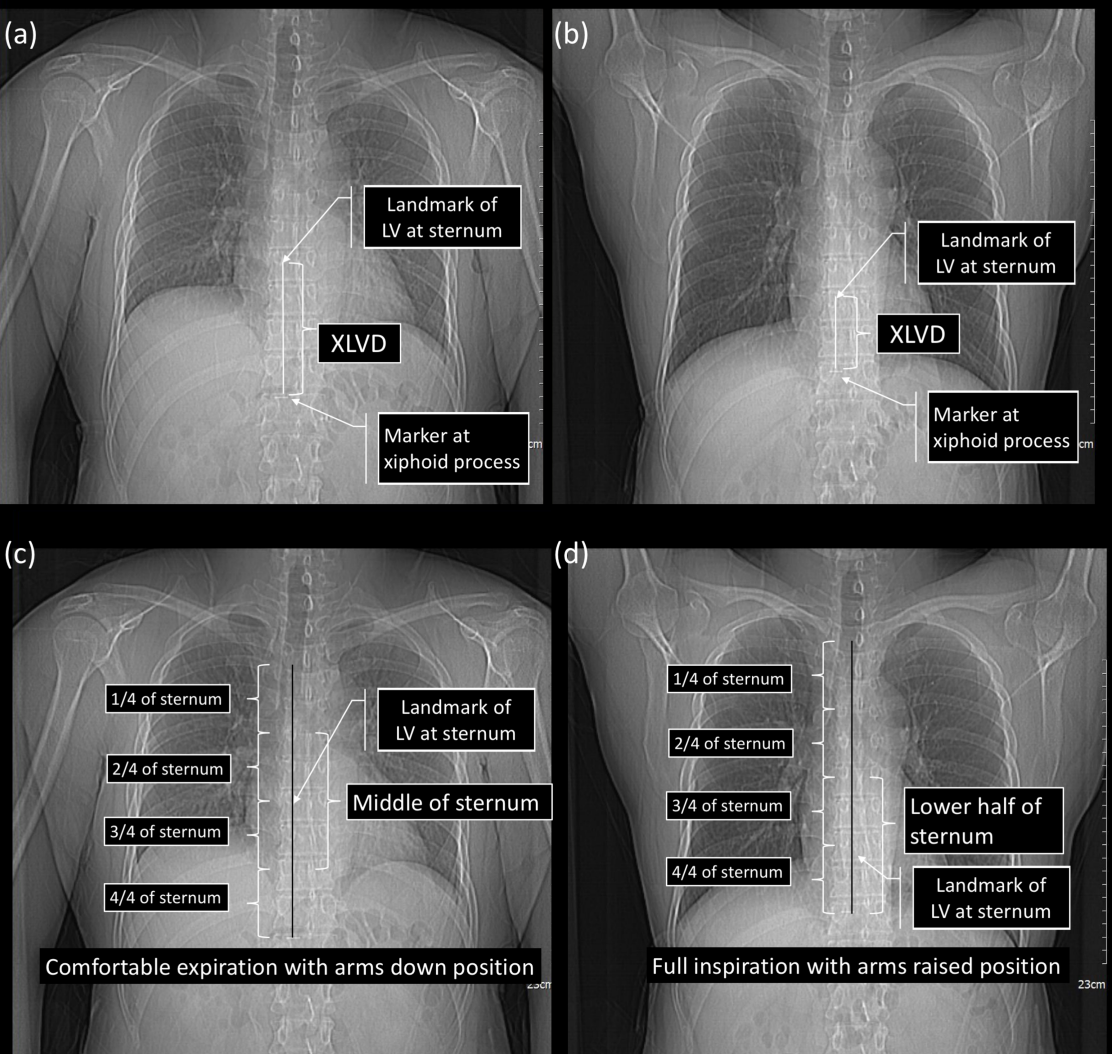
The distance from the xiphoid process to the point of the left ventricle’s maximal anteroposterior diameter of the left ventricle. The perpendicular line between the point for the left ventricle (LV)’s maximal anteroposterior diameter (APD) and the xiphoid process marker was measured in the expiration with the arms down position and the inspiration with the arms raised position (XLVD) (a,b). From the upper side of the sternum, the 1/4 and 2/4 sections were defined as the upper half of the sternum, the 2/4 and 3/4 sections were defined as the middle of the sternum, and the 3/4 and 4/4 sections were defined as the lower half of the sternum, relative to the positions of the heart and liver (c,d). In the expiration with the arms down position, the land marks of LV at sternum were located beneath the middle of the sternum, and the liver was located beneath the lower half of the sternum. In the inspiration with the arms raised position, the land marks of LV at sternum were located beneath the lower half of the sternum.

The sternum was divided into three zones (upper half, middle, and lower half) to define the relative positions of the heart, great vessels, and liver underneath the sternum based on the EAD scout view. The upper half, middle, and lower half of the sternum were defined as the 1/4 and 2/4 sections of the sternum, the 2/4 and 3/4 sections of the sternum, and the 3/4 and 4/4 sections of the sternum, respectively. The XLVD was measured on the EAD and IAR scout images, as well as the whole sternum length and the XLVD/sternum length, to compare the relative positions of the LV’s maximal APD in the EAD and IAR positions (Fig 3).

### Outcomes

The primary outcome was the evaluation of the LV’s location based on the sternal landmark using a comparison of the EAD and IAR scout images. The secondary outcome was the comparison of the organs under the three zones of the sternum between the EAD and IAR positions.

### Statistical analysis

Data were summarized as median and interquartile range for non-parametric continuous variables, as mean ± standard deviation for normally distributed parametric variables, and as frequency and percentage for categorical variables [11]. Data were compared using the Student t test for parametric variables, the paired t test for paired parametric variables, and the Wilcoxon rank-sum test for non-parametric continuous variables. Fisher’s exact test was used for categorical data. Univariable and multivariable logistic regression was conducted for accounting factors associated with the position of the heart according to respirations and positions of arms. All analyses were performed using STATA software (version 13.1; STATA Corp LP, College Station, TX, USA). A *P*-value of <0.05 was used to determine statistical significance.

## Results

### Participant characteristics

During the 5-month study period, 128 participants underwent LDCT and 1 participant was excluded because of an unidentifiable radio-opaque marker. Compared to the female participants, the male participants exhibited significantly higher values for height, weight, body mass index, and sternum length; no significant difference was observed for age (Table 1).

**Table 1:** Participant characteristics.

	All participants	Men	Women	*P*-value*
**n (%)**	127 (100)	59 (46)	68 (54)	
**Age (years),** **median (IQR)**	49 (43–53)	49 (40–53)	49 (43.5–52)	0.95
**Height (cm),** **mean (SD)**	165.1 (8.2)	171.7 (6.1)	159.4 (4.7)	<0.001
**Weight (kg),** **median (IQR)**	63.3 (57.4–70.9)	70.4 (65.9–76.3)	58.1 (43.2–63)	<0.001
**BMI,** **median (IQR)**	23.53 (21.6–25.04)	23.99 (22.72–25.26)	22.23 (21.145–24.045)	<0.001
**Sternum length (mm),** **median (IQR)**	181.9 (170.46–192.87)	188.7 (175.3–202.38)	177.67 (168.58–185.31)	<0.001

* *P*-value from the comparison of men and women.

BMI, body mass index; IQR, interquartile range; SD, standard deviation

### Locations of LV in the EAD and IAR positions

When the sternum was divided into the three zones, LV was located in the middle of the sternum at EAD for 117 participants (92%) and in the lower half of the sternum at IAR for 107 participants (84%). The XLVD and XLVD/sternum length values in the EAD position were >2-fold greater than those in the IAR position for all participants. There was no significant sex-related difference in the XLVD using the EAD position (*P* = 0.35). The XLVD/sternum length value was significantly higher among female participants, compared to male participants (*P* = 0.013). The XLVD and XLVD/sternum length values in the IAR position were significantly higher among female participants, compared to male participants (*P* < 0.05) (Table 2).

**Table 2:** Comparing distances from the xiphoid process to the sternum landmark according to respiratory phase and arm position.

		EAD image	SE	IAR image	SE	*P*-value
**XLVD (mm), mean (SD)**	Total	85.05 (20.64)	1.83	32.71 (17.33)	1.53	<0.001
	Men	83.21 (21.49)	2.79	27.91 (16.09)	2.09	<0.001
	Women	86.66 (19.90)	2.41	36.88 (17.40)	2.11	<0.001
	*P*-value*	0.35		0.003		
**XLVD/sternum length (%), mean (SD)**	Total	46.29 (10.08)	0.89	17.64 (8.78)	0.77	<0.001
	Men	43.91 (10.28)	1.34	14.40 (7.62)	0.99	<0.001
	Women	48.35 (9.50)	1.15	20.45 (8.79)	1.07	<0.001
	*P*-value*	0.013		<0.001		

* *P*-value from the comparison of men and women.

EAD: expiration with the arms down position; IAR: inspiration with the arms raised position; SE: standard error; XLVD: the distance from the xiphoid process to the sternum landmark for maximal anteroposterior diameter of the left ventricle.

### Locations of the organs under the three parts of the sternum

The liver’s left lobe was located in the lower half of the sternum at EAD for 118 participants (93%) (Table 3).

**Table 3:** Locations of the heart and liver according to the three sternum divisions.

Parts of the sternum	EAD, n (%)	SE	Heart IAR, n (%)	SE	*P*-value	EAD, n (%)	SE	Liver IAR, n (%)	SE	*P*-value
Upper half	1 (0.79)	1	0 (0)	-		0 (0)	-	0 (0)	-	
Middle	117 (92.13)	3.05	20 (15.75)	4.12	<0.001	0 (0)	-	0 (0)	-	<0.001
Lower half	9 (7.09)	2.90	107 (84.25)	4.12		118 (92.91)	2.90	20 (15.75)	4.12	

EAD: expiration with the arms down position; IAR: inspiration with the arms raised position; SE: standard error.

Upper half of the sternum = upper 1/4 of the sternum + 2/4 of the sternum

Middle of the sternum = 2/4 of the sternum + 3/4 of the sternum

Lower half of the sternum = 3/4 of the sternum + 4/4 of the sternum

### Factors associated with the location of the heart according to respiration and arms position

Logistic regression was performed to approach the correlation between biometrical covariates and the location of the heart according to respiration and arms position (mean variance inflation factor = 38.43) (Table 4).

**Table 4:** Univariable and multivariable analysis of factors associated with the location of the heart according to expiration and inspiration.

Location of the heart	Middle of the sternum in EAD					
	Univariable logistic regression			Multivariable logistic regression*		
	OR	SE	*P* value	OR	*SE*	*P value*
Age	0.95	0.04	*0*.*31*	0.96	*0*.*04*	*0*.*43*
Sex	0.19	0.16	0.04	0.02	0.03	0.006
Body weight	1.00	0.03	0.90	0.68	0.32	0.41
Height	0.99	0.04	0.81	1.56	0.60	0.24
Body mass index	1.05	0.13	0.69	3.63	4.85	0.34
Length of the sternum	0.99	0.02	0.67	0.99	0.02	0.65
Location of the heart	Lower half of the sternum in IAR					
	Univariable logistic regression			Multivariable logistic regression**		
	OR	SE	*P* value	OR	SE	*P* value
Age	0.95	0.04	0.31	1.03	0.04	0.50
Sex	3.06	1.69	0.04	2.61	2.69	0.35
Body weight	1.04	0.03	0.15	2.21	1.35	0.20
Height	0.99	0.04	0.81	0.65	0.30	0.34
Body mass index	1.04	0.09	0.66	0.12	0.18	0.17
Length of the sternum	0.96	0.01	0.001	0.90	0.02	<0.001

EAD: expiration with the arms down position; IAR: inspiration with the arms raised position; OR: odds ratio; SE: standard error.

* Hosmer–Lemeshow goodness-of-fit p-value = 0.69.

** Hosmer–Lemeshow goodness-of-fit p-value = 0.87.

Upper half of the sternum = upper 1/4 of the sternum + 2/4 of the sternum

Middle of the sternum = 2/4 of the sternum + 3/4 of the sternum

Lower half of the sternum = 3/4 of the sternum + 4/4 of the sternum

Sex might influence the position of the heart (Odds ratio = 0.02, *P* value = 0.006 in multivariable logistic regression) in EAD. However, only six males’ hearts (10% in males) and three felmales’ hearts (4% in females) were located in lower half of the sternum in EAD. Length of sternum might influence the position of the hearts in IAR but not in EAD.

## Discussion

This is the first CT study to investigate the LV’s location according respiration state and arms’ position. The recent CPR guidelines have recommended the lower half of the sternum as the site for proper hand positioning during chest compressions, in order to effectively compress the LV. However, the lower half of the sternum is an ambiguous point, and it was recommended because of its easy applicability and teachability, rather than because of specific evidence that it effectively generates cardiac output [12, 13]. According to the results of the present study, the lower half of the sternum is appropriate for compressing the LV if the IAR position is maintained during CPR, but this is unrealistic, as patients typically receive CPR in the EAD position. Thus, the middle of the sternum might be the optimal hand position for effectively compressing the LV and avoiding direct compression of the liver’s left lobe.

The majority of recent evidence has identified the LV’s location for chest compressions based on CT images, and has supported the current CPR guidelines. In a previous study of chest CT images from 189 patients, the root of the aorta, the ascending aorta, and the left ventricular outflow tract were located immediately below the midpoint of the inter-nipple line in 80% of patients and 20% of the left ventricles were located below the midpoint of the inter-nipple line, which suggested that chest compressions at the end of the sternum might be more efficient than compressions at the midpoint of the inter-nipple line [10]. Another study revealed that the most commonly compressed anatomical structures were the ascending aorta and the top of the left atrium [8]. Furthermore, the inter-nipple line was not an optimal chest compression point in another study’s paediatric population [14]. Nevertheless, all of these studies were based on chest CT that was taken in the IAR position, which may not reflect real-life CPR.

Chest compressions are associated with several significant complications, and even with adequate CPR execution [15, 16]. For example, the liver is a major organ that might be compressed during CPR if the lower half of the sternum is compressed, and the liver’s left lobe is affected in 0.6–2.1% of autopsy cases, because of its anatomical position [15, 17]. However, given that most non-survivors do not undergo autopsy, and not all survivors undergo abdominal CT or other evaluations, the number of liver injuries may be underestimated. In addition, injuries to the visceral structures are mainly related to fractured ribs and/or a fractured sternum. In a recent study, the middle and lower third parts of the sternum were the most common fracture sites among cases of CPR complications [16]. Moreover, compressions at the lower half of the sternum have a higher risk of fracturing the xiphoid process during CPR, which can also injure the liver. Thus, higher hand positioning, and avoiding compressions on the lowest part of the sternum, may help prevent liver injury and sternal fracture. Nevertheless, the sternum acts as a hinge that is fixed superior to the thoracic inlet, and compressions typically move the lower part of the sternum, based on chest CT results [8]. Therefore, compressions at the middle of the sternum might make it difficult for rescuers to achieve appropriate compression depth, compared to compressions at the lower half of the sternum.

This study had several limitations. First, the study has limited external validity because it was intentionally performed in a relatively healthy population. Second, we did not collect axial CT images during expiration, which prevented us from precisely evaluating the structures and organs that were compressed at each position. Third, uncertainty regarding whether the heart was in systole or diastole might produce bias in the measurement of the heart’s centre. Fourth, comfortable expiration could be considered ambiguous, and expiration might be different for each participant. Fifth, we did not test the hemodynamic effect of chest compressions, and further interventional trials are needed to address this topic. Finally, male and female participants exhibited different XLVD values (approximately 5 mm) and XLVD/sternum length values (approximately 5%), although these relatively small difference are likely irrelevant during real-life CPR. Similarly, there were no sex-specific differences in the intra-thoracic organ locations under the sternum.

## Conclusions

The present study’s results indicate that the middle of the sternum might be the optimal location for targeting the LV during CPR and the liver’s left lobe might be located in the lower half of the sternum during CPR.

## Supporting information

S1 File Supporting data of enrolled patients and positions of their left ventricles and organs. (PDF)
