# Supplementary material for: Where is the left ventricle during cardiopulmonary resuscitation based on chest computed tomography in the expiration with arms down position?
Source: PLoS One. 2018 Feb 23;13(2):e0193364. doi: 10.1371/journal.pone.0193364 (PMC5825080; doi:10.1371/journal.pone.0193364)
Supplement: S1 File — (PDF) [file pone.0193364.s001.pdf]

age (year)	sex	body weight (kg)	height (cm)	body nass index	length of sternum	XLVD_expiration
48.00	F	58.00	162.00	22.10	187.99	83.02
45.00	F	66.50	163.00	25.03	225.13	149.91
37.00	F	63.90	158.00	25.60	163.09	75.71
29.00	F	58.10	164.00	21.60	228.52	124.02
49.00	F	55.60	162.00	21.19	192.88	98.65
50.00	F	58.10	164.00	21.60	179.20	69.34
50.00	F	52.30	154.00	22.05	170.43	82.52
49.00	F	39.70	152.00	17.18	163.68	71.30
44.00	F	45.60	158.00	18.27	209.49	91.34
37.00	F	61.50	161.00	23.73	181.28	87.90
36.00	F	63.00	169.00	22.06	216.68	113.28
49.00	F	56.80	164.00	21.12	185.06	68.85
43.00	F	50.10	154.00	21.12	182.63	98.65
43.00	F	44.40	161.00	17.13	181.26	77.02
32.00	F	54.80	159.00	21.68	184.08	88.87
54.00	F	57.70	156.00	23.71	159.67	78.61
51.00	F	51.60	162.00	19.66	162.95	99.61
43.00	F	51.50	159.00	20.37	189.94	72.29
37.00	F	53.20	164.00	19.78	175.90	102.05
45.00	F	70.50	162.00	26.86	188.15	83.99
57.00	F	75.80	158.00	30.36	156.25	91.31
42.00	F	59.20	166.00	21.48	160.65	60.06
54.00	F	58.20	156.00	23.92	165.44	68.91
57.00	F	54.90	152.00	23.76	180.32	81.06
49.00	F	52.60	156.00	21.61	173.44	67.87
40.00	F	58.20	162.00	22.18	203.62	106.45
52.00	F	66.70	152.00	28.87	178.71	108.89
45.00	F	54.20	160.00	21.17	181.65	60.58
39.00	F	50.80	165.00	18.66	201.18	102.58
42.00	F	47.60	156.00	19.56	168.58	93.29
52.00	F	53.10	153.00	22.68	180.19	111.37
55.00	F	59.40	162.00	22.63	168.51	101.08
45.00	F	58.30	157.00	23.65	170.90	86.92
41.00	F	57.40	164.00	21.34	187.50	108.44
52.00	F	59.50	159.00	23.54	155.84	74.71
52.00	F	86.50	160.00	33.79	169.49	73.74
55.00	F	59.10	162.00	22.52	178.74	92.29
59.00	F	67.20	168.00	23.81	179.18	127.63
57.00	F	48.70	148.00	22.23	174.90	78.14
39.00	F	49.00	164.00	18.22	177.46	90.98
52.00	F	59.60	160.00	23.28	153.82	22.95
49.00	F	63.00	157.00	25.56	158.69	85.48
58.00	F	74.20	155.00	30.88	166.51	85.97
49.00	F	55.40	160.00	21.64	182.67	93.77
58.00	F	63.30	161.00	24.42	172.85	93.26
56.00	F	67.80	158.00	27.16	164.08	82.05
44.00	F	61.60	165.00	22.63	167.58	80.08
50.00	F	63.60	158.00	25.48	171.06	89.84
50.00	F	60.30	156.00	24.78	164.11	75.78
50.00	F	45.80	149.00	20.63	173.35	47.87
51.00	F	54.80	156.00	22.52	177.87	53.26
50.00	F	55.30	165.00	20.31	187.01	83.98
51.00	F	46.90	156.00	19.27	170.46	60.07
38.00	F	54.60	158.00	21.87	172.94	69.34
51.00	F	53.30	162.00	20.31	184.62	76.17

44.00	F	68.10	169.00	23.84	184.13	116.21
50.00	F	66.10	155.00	27.51	172.86	88.93
51.00	F	65.30	159.00	25.83	168.58	95.73
46.00	F	66.30	169.00	23.21	183.59	91.80
54.00	F	55.00	161.00	21.22	163.12	60.08
43.00	F	50.70	157.00	20.57	174.40	94.24
45.00	F	48.60	157.00	19.72	185.56	91.31
49.00	F	54.80	157.00	22.23	185.71	92.77
44.00	F	58.40	154.00	24.62	170.41	77.64
54.00	F	70.70	159.00	27.97	190.03	100.12
48.00	F	58.50	156.00	24.04	193.86	122.59
50.00	F	59.40	167.00	21.30	192.40	73.74
51.00	F	56.30	153.00	24.05	169.43	84.47
46.00	M	81.20	175.00	26.51	172.42	103.03
50.00	M	67.50	170.00	23.36	167.98	62.99
47.00	M	82.60	174.00	27.28	207.14	80.57
52.00	M	67.00	162.00	25.53	183.11	85.94
43.00	M	67.40	162.00	25.68	172.85	86.92
40.00	M	64.00	176.00	20.66	175.79	88.87
53.00	M	62.60	166.00	22.72	206.06	129.39
22.00	M	72.80	176.00	23.50	215.82	85.45
57.00	M	73.00	170.00	25.26	223.15	105.47
65.00	M	62.50	162.00	23.81	176.32	39.55
56.00	M	72.70	176.00	23.47	215.36	135.25
47.00	M	69.90	176.00	22.57	192.87	87.90
53.00	M	75.80	174.00	25.04	191.43	69.34
54.00	M	77.50	182.00	23.40	209.96	101.56
54.00	M	60.20	166.00	21.85	198.79	78.16
57.00	M	68.60	170.00	23.74	188.70	53.71
63.00	M	78.50	173.00	26.23	200.22	131.36
58.00	M	59.30	172.00	20.04	183.59	24.90
35.00	M	65.80	167.00	23.59	211.43	93.26
46.00	M	66.30	177.00	21.16	218.76	77.71
42.00	M	61.80	171.00	21.13	196.78	104.98
34.00	M	70.30	165.00	25.82	207.04	115.81
45.00	M	70.00	166.00	25.40	186.52	98.14
40.00	M	62.60	167.00	22.45	171.88	86.43
40.00	M	71.80	173.00	23.99	210.50	86.91
46.00	M	76.30	171.00	26.09	175.30	114.76
29.00	M	77.60	177.00	24.77	180.73	85.94
52.00	M	65.90	167.00	23.63	195.32	97.17
21.00	M	122.70	185.00	35.85	185.56	88.38
56.00	M	80.50	179.00	25.12	185.06	73.40
39.00	M	62.50	171.00	21.37	163.16	48.83
52.00	M	73.80	177.00	23.56	215.33	89.36
40.00	M	62.00	161.00	23.92	179.22	84.49
51.00	M	74.50	167.00	26.71	172.40	78.61
53.00	M	70.90	167.00	25.42	182.64	54.20
43.00	M	57.40	163.00	21.60	202.38	72.27
38.00	M	80.30	181.00	24.51	230.02	106.97
42.00	M	68.50	176.00	22.11	200.20	76.67
53.00	M	73.60	171.00	25.17	227.05	80.57
51.00	M	73.40	171.00	25.10	185.09	61.04
50.00	M	76.30	184.00	22.54	194.44	76.74
56.00	M	84.30	180.00	26.02	185.60	69.34
43.00	M	67.00	163.00	25.22	146.00	46.88

53.00	M	76.10	178.00	24.02	188.78	75.71
50.00	M	70.90	172.00	23.97	188.96	91.33
59.00	M	59.70	161.00	23.03	165.63	93.76
39.00	M	70.40	170.00	24.36	200.71	97.66
50.00	M	66.80	170.00	23.11	165.63	93.76
57.00	M	73.20	173.00	24.46	184.08	88.87
35.00	M	81.50	183.00	24.34	211.93	89.84
58.00	M	66.80	166.00	24.24	188.97	85.46
35.00	M	65.60	171.00	22.43	200.21	74.00
33.00	M	82.30	182.00	24.85	191.44	67.40
54.00	M	68.00	170.00	23.53	136.29	53.24
52.00	M	77.30	164.00	28.74	166.50	71.29
49.00	M	66.30	171.00	22.67	162.18	66.44
45.00	M	74.80	175.00	24.42	181.90	89.86
43.00	M	57.70	175.00	18.84	166.51	53.73
37.00	M	85.50	170.00	29.58	171.52	97.66

XLVD: the distance from the xiphoid process to the sternum landmark for maximal anteroposteri[illegible]

v: vessel, h: heart, l: liver

XLVD_inspiration	expiration_1/4ofsternum	expiration_2/4ofsternum	expiration_3/4ofsternum
41.55	v	h	h
81.06	v	h	l
27.34	v	h	l
83.50	v	h	l
41.03	v	h	l
39.09	v	h	l
47.87	v	h	l
24.42	v	h	h
57.69	v	h	h
37.12	v	h	h
52.79	v	h	l
33.72	v	h	h
44.46	v	h	l
27.00	v	h	h
58.24	v	h	h
37.11	v	h	l
44.48	v	h	l
25.41	v	h	l
46.40	v	h	l
53.71	v	h	h
32.22	v	h	l
23.93	v	h	h
20.54	v	h	h
55.74	v	h	h
30.77	v	h	h
48.38	v	h	l
49.81	v	h	l
24.91	v	h	h
63.97	v	h	h
43.95	v	h	l
42.99	v	h	l
33.20	v	h	l
14.66	v	h	l
40.04	v	h	l
27.83	v	h	l
29.30	v	h	l
52.73	v	h	l
40.21	v	h	l
33.72	v	h	h
31.74	v	h	h
7.32	v	h	h
27.83	v	h	l
30.76	v	h	l
47.87	v	h	l
43.95	v	h	h
24.95	v	h	h
14.17	v	h	h
35.16	v	h	l
32.29	v	h	h
28.81	v	v	h
41.02	v	h	h
17.58	v	h	h
12.22	v	h	h
15.63	v	h	h
21.00	v	h	h

88.87	v	h	l
15.17	v	h	l
34.18	v	h	l
12.25	v	h	l
5.88	v	h	h
37.61	v	h	l
48.84	v	h	l
37.61	v	h	l
22.46	v	h	l
32.75	v	h	l
68.85	h	h	l
7.32	v	h	h
50.79	v	h	l
34.68	v	v	h
36.62	v	v	h
17.10	v	h	h
18.70	v	h	h
27.35	v	h	l
52.11	v	h	h
54.20	v	h	l
38.09	v	h	h
33.69	v	v	h
9.77	v	v	h
74.72	v	h	l
19.53	v	h	h
9.28	v	h	h
40.53	v	h	l
21.00	v	h	l
13.71	v	v	h
44.44	v	h	l
1.95	v	h	h
54.69	v	h	l
20.51	v	h	h
39.55	v	v	h
54.72	v	h	l
32.23	v	h	l
22.48	v	h	h
46.88	v	h	h
33.21	v	h	h
14.16	v	h	l
41.50	v	h	h
21.02	v	h	h
22.46	v	h	h
3.42	v	v	h
38.09	v	h	h
30.29	v	h	l
28.81	v	h	l
15.63	v	h	h
15.14	v	v	h
51.33	v	h	h
53.71	v	h	h
50.78	v	h	h
25.11	v	h	h
25.41	v	h	h
7.32	v	h	h
1.46	v	h	h

27.38	v	h	h
28.34	v	h	l
16.12	v	h	l
44.44	v	h	h
16.12	v	h	l
11.76	v	h	l
24.91	v	h	h
26.86	v	h	l
45.96	v	h	h
13.71	v	h	h
12.76	v	h	l
22.48	v	h	l
11.73	v	h	h
5.88	v	h	l
13.19	v	h	h
27.83	v	h	l

teroposterior diameter of the left ventricle.

expiration_4/4ofsternum	inspiration_1/4ofsternum	inspiration_2/4ofsternum	inspiration_3/4ofsternum
l	v	v	h
l	v	h	h
l	v	v	h
l	v	h	h
l	v	h	h
l	v	v	h
l	v	h	h
l	v	v	h
l	v	h	h
l	v	v	h
l	v	v	h
l	v	h	h
l	v	h	h
l	v	v	h
l	v	h	h
l	v	v	h
l	v	h	h
l	v	h	h
l	v	h	h
l	v	h	h
l	v	h	h
l	v	v	h
l	v	v	h
l	v	h	h
l	v	v	h
l	v	h	h
l	v	h	h
h	v	v	h
l	v	h	h
l	v	h	h
l	v	v	h
l	v	v	h
l	v	v	h
l	v	v	h
l	v	v	h
l	v	v	h
l	v	h	h
l	v	h	h
l	v	v	h
l	v	v	h
h	v	v	h
l	v	v	h
l	v	v	h
l	v	v	h
l	v	h	h
l	v	v	h
l	v	v	h
l	v	v	h
l	v	v	h
h	v	v	h
l	v	v	h
l	v	v	h
l	v	v	h
l	v	v	h
l	v	v	h

l	v	h	l
l	v	v	h
l	v	v	h
l	v	v	h
l	v	v	h
l	v	v	h
l	v	v	h
l	v	v	h
l	v	v	h
l	v	v	h
l	v	h	h
l	v	v	h
l	v	h	h
h	v	v	h
h	v	v	h
l	v	v	h
l	v	v	h
l	v	v	h
l	v	v	h
l	v	v	h
l	v	v	h
l	v	v	h
h	v	v	h
l	v	h	h
l	v	v	h
l	v	v	h
l	v	v	h
l	v	v	h
h	v	v	h
l	v	v	h
h	v	v	h
l	v	v	h
l	v	v	h
l	v	v	h
l	v	v	h
l	v	v	h
l	v	v	h
l	v	v	h
l	v	v	h
l	v	v	h
l	v	v	h
l	v	v	h
l	v	v	h
l	v	v	h
l	v	v	h
l	v	v	h
l	v	v	h
l	v	v	h
l	v	v	h
l	v	v	h
l	v	h	h
l	v	v	h
l	v	v	h
l	v	v	h
l	v	v	h
l	v	v	h

l	v	v	h
l	v	v	h
l	v	v	h
l	v	v	h
l	v	v	h
l	v	v	h
l	v	v	h
l	v	v	h
h	v	v	h
l	v	v	h
l	v	v	h
l	v	v	h
l	v	v	h
l	v	v	h
l	v	v	h
l	v	v	h

inspiration_4/4ofsternum
h
l
h
l
h
h
h
h
l
h
h
h
l
h
l
h
h
h
h
l
h
h
h
l
h
l
l
h
l
h
h
h
h
h
h
h
l
h
h
h
h
h
h
h
l
h
h
h
h
h
h
h
h
h
h

l
h
h
h
h
h
h
h
h
h
l
h
l
h
h
h
h
h
h
l
h
h
h
l
h
h
h
h
h
h
h
l
h
h
l
h
h
h
h
h
h
h
h
h
h
h
h
h
h
h
l
h
h
h
h
h

h
h
h
h
h
h
h
h
h
h
h
h
h
h
h
h
